# Medical Degree Disparity Among Authors of Original Research in Pediatric Journals: A Four-Year Follow-Up

**DOI:** 10.7759/cureus.9050

**Published:** 2020-07-07

**Authors:** Michael Dabrowski, John Ashurst

**Affiliations:** 1 Medicine, Arizona College of Osteopathic Medicine, Glendale, USA; 2 Emergency Medicine, Kingman Regional Medical Center, Kingman, USA

**Keywords:** osteopathic, pediatrics, allopathic, scholarly acrtivity, publication

## Abstract

Introduction and Objective: Scholarly activity is a major component of residency training and the accreditation process for graduate medical education. In 2014, Accreditation Council for Graduate Medical Education and the American Association of Colleges of Osteopathic Medicine announced a single accreditation system with the transition beginning July 1, 2015. Previous data before the transition had shown that osteopathic physicians rarely published original research in three high-impact pediatric journals. The objective of this study is to determine if there is a degree disparity between osteopathic and allopathic physicians among authors who publish original research manuscripts in three high-impact pediatric journals after the beginning of the transition to a single graduate medical education accreditation system.

Methods: Degree designation for the first and senior authors of original research manuscripts was reviewed for the Journal of Pediatrics (J Pediatr), Pediatrics, and JAMA Pediatrics (JAMA Pediatr) for the years 2016, 2017, 2018, and 2019. Inter-rater reliability was calculated by the kappa coefficient, and data were analyzed with descriptive statistics and simple linear regression.

Results: A total of 3,252 manuscripts and 4,068 authors were reviewed with 0.98% of all authors being osteopathic physicians. A total of 1.65% of first authors and 0.41% of senior authors were osteopathic physicians. For those with a dual degree, a total of 1.03% of first, and 0.41% of senior authors were osteopathic physicians. No statistical trend could be established for increased first, senior, dual-degree first, or dual-degree senior osteopathic physician authorship.

Conclusion: Osteopathic physicians continue to be underrepresented as first and senior authors in original publications in the three high-impact pediatric journals as compared to their allopathic counterparts.

## Introduction

In February 2014, the Accreditation Council for Graduate Medical Education (ACGME) and the American Association of Colleges of Osteopathic Medicine announced a single accreditation system for graduate medical education. In this announcement, traditional osteopathic residencies were allowed to apply for ACGME accreditation beginning July 1, 2015 and all residencies must have received initial accreditation by July 30, 2020 [1]. With the transition to a single accreditation system, the requirements for scholarly activity have undergone a dramatic shift from being resident focused to now being faculty driven. 

Prior to the merger, the American Osteopathic Association stated that osteopathic residents in a pediatric residency “had to complete one scientific scholarly writing project” that had to be submitted for publication [2]. However, the ACGME notes that residents must participate in scholarship but core faculty must demonstrate scholarly activity by either receiving a peer-reviewed grant, research in the basic sciences, education, translational science, patient care or population health, quality improvement or patient safety initiatives, systematic reviews, creation of curricula or educational materials, innovation of education, or contribution to professional committees [3].

Recent data have shown that meeting several of these scholarly activity requirements as set forth by the ACGME could be difficult for osteopathic physicians. In the specialties of emergency medicine, general surgery, and family medicine, no osteopathic physician has received a National Institutes of Health R01 grant in more than a decade [4-6]. In a recent study examining the scholarly activity of osteopathic physicians prior to the merger, very few served as either the first or senior author on research that had been published in high-impact pediatric journals [7]. However, no data exist on the publication patterns of pediatricians since programs began to transition during the single accreditation system. The authors sought to determine a medical degree disparity among physician-scientists who published manuscripts in three high-impact pediatric journals in 2016, 2017, 2018, and 2019. 

## Materials and methods

Study design

Following institutional review board approval, a retrospective analysis of medical degree trends in original research publications from the Journal of Pediatrics (J Pediatr), Pediatrics (Pediatrics), and JAMA Pediatrics (JAMA Pediatr) was undertaken for the years 2016, 2017, 2018, and 2019. Medical degree was determined by examining the first and last (senior) authors of each manuscript. Authors were categorized as either having an allopathic (MD) or osteopathic (DO) degree in medicine. Only original research manuscripts were included and all other types of manuscripts including case reports, clinical images, reviews, commentaries, and letters to the editor were excluded from final analysis. Manuscripts not authored by a physician were also excluded from analysis. For those with more than one advanced degree, both the medical degree and advanced degree (MPH, MSc, PhD, etc.) were reviewed. Peer review training in proper abstracting technique was conducted by the senior author by reviewing the authors and degrees for the first month of Pediatrics. After data collection, 100 total data points were chosen by a random number generator to determine inter-rater reliability.

Data snalysis

Descriptive statistics was used to compare the proportions of MD and DO authorship and for those holding both a medical degree and a second advanced degree across the years studied. Trends in authorship were analyzed using simple linear regression, and inter-rater reliability was determined using the kappa coefficient. Statistical significance was defined as a P≤0.05.

## Results

A total of 3,252 manuscripts and 4,068 authors were reviewed for the years included in analysis. A total of 1,874 authors were considered first authors and of these 874 authors held a dual degree. A total of 2,194 authors were considered the senior authors on a manuscript with 1,263 of these authors holding a dual degree. Inter-rater reliability for first and senior author degree designation was K=1. 

Overall, 0.98% of all authors (40/4,068) were osteopathic physicians, while 99.02% (4,028/4,068) were allopathic physicians. When only first authors were considered, a total of 1.65% (31/1874) were osteopathic physicians and 98.35% (1,843/1874) were allopathic physicians. For those authors who held a dual degree and were the first authors of a manuscript, only 1.03% (9/874) were osteopathic physicians. When the last author was considered, 0.41% (9/2,194) of physicians held a degree in osteopathic medicine and the remainder were allopathic physicians. Similarly, 0.24% (3/1,263) of those senior authors holding a dual degree were also osteopathic physicians. 

When all journals reviewed were considered, no statistically significant trend could be established for increased first or senior author publications (Table 1). However, a statistically significant negative trend (p=0.01) for DO first author publications was noted for the J Pediatr. A statistically significant trend was also noted for first (P=0.033) and senior (P=0.014) MD authors who published in Pediatrics. 

**Table 1:** Percentage of DOs and MDs Serving as First or Senior Author by Journal and Year J Pediatr, Journal of Pediatrics; DO, osteopathic physician; JAMA Pediatr, JAMA Pediatrics; MD, allopathic physician. P values were calculated using linear regression analyses. Findings were statistically significant at P≤0.05.

Authorship by Journal	DO or MD as First or Senior Author				P Value
	2016	2017	2018	2019	
Overall					
DO authors					
First	2.02 (10/494)	2.05 (10/488)	1.03 (5/486)	1.48 (6/406)	0.16
Senior	0 (0/586)	0.36 (2/560)	0.70 (4/568)	0.63 (3/480)	0.16
MD authors					
First	97.98 (484/494)	97.95 (478/488)	98.97 (481/486)	98.52 (400/406)	0.21
Senior	100 (586/586)	99.64 (558/560)	99.30 (564/568)	99.37 (477/480)	0.13
J Pediatr					
DO authors					
First	2.69 (7/260)	1.98 (5/253)	1.04 (3/288)	0.92 (2/218)	0.01
Senior	0 (0/307)	0 (0/297)	0.61 (2/328)	0.74 (2/272)	0.11
MD authors					
First	97.31 (253/260)	98.02 (248/253)	98.96 (285/288)	99.08 (216/218)	0.66
Senior	100 (307/307)	100 (297/297)	99.39 (326/328)	99.26 (270/272)	0.54
Pediatrics					
DO authors					
First	1.65 (3/182)	2.33 (4/172)	1.38 (2/145)	2.11 (3/142)	0.68
Senior	0 (0/215)	1.04 (2/193)	1.09 (2/184)	0.63 (1/158)	0.60
MD authors					
First	98.35 (179/182)	97.67 (168/172)	98.62 (143/145)	97.89 (139/142)	0.033
Senior	100 (215/215)	98.96 (191/193)	98.91 (182/184)	99.37 (157/158)	0.014
JAMA Pediatr					
DO authors					
First	0 (0/52)	1.59 (1/63)	0 (0/53)	2.17 (1/46)	0.55
Senior	0 (0/64)	0 (0/70)	0 (0/56)	0 (0/50)	
MD authors					
First	100 (52/52)	98.41 (62/63)	100 (53/53)	97.83 (45/46)	0.44
Senior	100 (64/64)	100 (70/70)	100 (56/56)	100 (50/50)	0.18

For those DO authors with a dual degree, no statistically significant trend could be established for either first or senior authors when all journals reviewed were considered (Table 2). Likewise, no statistically significant trend could be established for DO authors with a dual degree in any individual journal reviewed. However, a statistically significant trend was noted for MD first authors (P=0.0033) who held a dual degree when all journals were considered. A significant trend was also seen for senior MD authors who held a dual degree who published manuscripts in the J Pediatr.

**Table 2:** Percentage of DOs and MDs With Dual Degrees Serving as First or Senior Author by Journal and Year J Pediatr, Journal of Pediatrics; DO, osteopathic physician; JAMA Pediatr, JAMA Pediatrics; MD, allopathic physician. P values were calculated using linear regression analyses. Findings were statistically significant at P≤0.05.

Authorship by Journal	DO or MD With Dual Degrees as First or Senior Author				P Value
	2016	2017	2018	2019	
Overall					
DO authors					
First	1.29 (3/232)	1.34 (3/224)	0.93 (2/215)	0.49 (1/203)	0.06
Senior	0 (0/325)	0.31 (1/327)	0.62 (2/320)	0 (0/291)	0.87
MD authors					
First	98.71 (229/232)	98.66 (221/224)	99.07 (213/215)	99.51 (202/203)	0.0033
Senior	100 (325/325)	99.69 (326/327)	99.38 (318/320)	100 (291/291)	0.13
J Pediatr					
DO authors					
First	1.09 (1/92)	2.27 (2/88)	0 (0/106)	1.10 (1/91)	0.68
Senior	0 (0/147)	0 (0/155)	0.59 (1/169)	0 (0/152)	0.74
MD authors					
First	98.91 (91/92)	97.73 (86/88)	100 (106/106)	98.90 (90/91)	0.77
Senior	100 (147/147)	100 (155/155)	99.41 (168/169)	100 (152/152)	0.61
Pediatrics					
DO authors					
First	1.87 (2/107)	1.02 (1/98)	2.44 (2/82)	0 (0/82)	0.33
Senior	0 (0/136)	0.77 (1/130)	0.88 (1/114)	0 (0/101)	1
MD authors					
First	98.13 (105/107)	98.98 (97/98)	97.56 (80/82)	100 (82/82)	0.076
Senior	100 (136/136)	99.23 (129/130)	99.12 (113/114)	100 (101/101)	0.0098
JAMA Pediatr					
DO authors					
First	0 (0/33)	0 (0/38)	0 (0/27)	0 (0/30)	
Senior	0 (0/42)	0 (0/42)	0 (0/37)	0 (0/38)	
MD authors					
First	100 (33/33)	100 (38/38)	100 (27/27)	100 (30/30)	0.45
Senior	100 (42/42)	100 (42/42)	100 (37/37)	100 (38/38)	0.16

## Discussion

As the current transition to a single graduate medical education accrediting system is reaching completion, based on our results, there still appears to be a significant gap in the number of publications when comparing osteopathic physicians with their allopathic counterparts in the pediatric specialty. These data are similar to the previous reported literature on pediatric DO publication patterns [7]. The cause of this persistent disparity in publications remains unclear, despite the accrediting bodies recommendations. However, it could be related to the demographics of the current workforce and training of DO physicians in pediatrics.

The osteopathic medical profession has historically focused on the education of non-subspecialist primary care physicians. Even though this trend has been declining over the past decade, there is still a significant number of DOs practicing primary care [8]. This focus on non-subspecialty primary care and its relationship to research attitudes could be a contributing factor to the authorship gap. Pediatric residents who expressed interest in pursuing non-subspecialty pediatrics were far more likely to report no interest in research than those who were interested in pursuing a subspecialty [9]. Another possible factor is that those in the non-subspecialty group of pediatric residents had fair or poor self-assessments of grant and manuscript writing [9].

Based on the data from the Association of American Medical Colleges before the transition to a single accreditation system in 2015, 4.45% of active physicians in pediatrics held a degree in osteopathic medicine and 11.27% of residents and fellows held a similar medical degree. By 2017, however, there appears to be an upward trend in active pediatric physicians and pediatric residents holding a degree in osteopathic medicine [10]. This reflects an increase in osteopathic physicians entering the field of pediatrics and could have a significant role in the future as the current osteopathic pediatric residents’ transition to core faculty roles in regards to the medical degree disparity in academic pediatrics. 

As the transition to a single accrediting system has not been fully completed, the final impact it may have on authorship trends cannot yet be clearly stated. Given the current accrediting standards, time to complete research, and time to publish completed research, it could take several more years to determine if a trend for increased DO publications occurs. 

Limitations

The data did not investigate MD vs DO authorship other than as senior and first author and cannot comment on any trends of authors with MD vs DO degrees outside of the senior and first authors placeholder. In addition, it is possible that the order of authorship in the articles evaluated did not follow the conventional listing of first author and senior author as main contributors and instead listed them alphabetically or in another fashion. Additionally, the data focused only on research in three high-impact pediatric journals and should not be applied to other medical specialties. The current research only looked at allopathic and osteopathic authors that published journal articles and does not explore if there is also a degree bias in the decision-making process of acceptance vs rejection of pediatric manuscripts. The current study also did not directly assess the total number of publications by residents or core faculty members of a residency prior to or following the transition to a single accreditation system. 

## Conclusions

Osteopathic physicians continue to be underrepresented as first and senior authors in original publications in the three high-impact pediatric journals that were evaluated during the time period of the ACGME transition to the single accreditation system for graduate medical education. Further research exploring the attitudes, training, and administrative roles of pediatric osteopathic physicians should be undertaken to determine the cause for the degree disparity in those who publish in pediatric journals. 
